# Book Reviews

**Published:** 1900-11

**Authors:** 


### Book Reviews.

A TREATISE ON APPENDICITIS. By JOHN B. DEAVER, M. D., Surgeon-in-Chief to the German Hospital, Philadelphia. Second edition. Thoroughly revised and considerably enlarged. Illustrated, with 32 full-page plates. Octavo. Philadelphia. P. Blakiston's Son & Co. 1900. [Price, $3.50, net.]

A new edition of this famous work is welcomed by the profession. Surgeons are always interested in the literature of this important malady, which is now generally recognised as essentially a surgical disease. Physicians, too—we mean those who do not practice surgery—are scarcely less interested, for, speaking generally, it is they who see the cases first and must early determine not only the time of operation, when operation becomes necessary, but who shall be the operator.

This treatise has been practically rewritten, since every chapter bears traces of new work and much additional material has been incorporated. As might be anticipated in a work by this author, it is particularly strong in its anatomical descriptions. Several hitherto undescribed landmarks for the surgeon are noted, and the plates, showing the appendix and its environs from several points of view, are simply superb. The section on pathology, too, deserves special mention. This, the author says, was prepared by Dr. A. O. J. Kelly, director of the pathologic institute of the German Hospital. The classic and aberrant forms of appendicitis are well described, including the catarrhal, tubercular, interstitial, ulcerative, carcinomatous and gangrenous varieties, together with their complications. Diffuse or generalised peritonitis and chronic appendicular peritonitis receive due attention, and their significance is well accentuated. The old fashioned "inflammation of the bowels" is no longer heard of as a pathologic entity, and these have taken its place, giving an intelligent expression of symptoms to correct pathologic bases; substituting, indeed, precision and certainty, for a former guesswork, which was almost always wrong.

The section on diagnosis is an attractive part of the book. It is especially interesting to the general practitioner. It informs him that ordinarily the diagnosis of this affection is unattended by special difficulty, but it also points out, by a carefully prepared dissertation on differential diagnosis, how he may arrive at a reasonable degree of certainty in doubtful or obscure or complicated cases. In treatment he is told that the most important immediate remedy is the exhibition of a laxative, whereas formerly it was supposed to be the first duty of the physician to administer an opiate. Here is a complete change of front in practice, based upon observations at the bedside and at the operating-table. If the symptoms are not relieved by laxatives it is time to begin preparations for surgical interference, and to be always ready, even in the presence of the most passive symptoms, to invoke the aid of the knife whenever the first danger signals are hoisted.

In his description of the several operations Deaver is clear and precise; the language used is free from high sounding phrases, but convinces the reader that the author is thoroughly familiar with his subject, and knows what he is writing about. He is teaching something that he has learned by experience, and announces conclusions reached by perhaps the largest series of operations of any surgeon in the world. A word should be spoken for the illustrations. The plates are some in black and some in colors, but all are specimens of an artistic excellence, an accuracy rarely seen. They add much to the intelligence as well as beauty of the volume, which, mechanically speaking, is beyond criticism.

This treatise may be regarded as the last word on appendicitis and represents the sum of our knowledge relating to that disease up to the moment of its issue. It is written by a master of the subject and reflects great credit upon American Surgery and American Surgeons.

---

THE TREATMENT OF FRACTURES. By CHARLES LOCKE SCUDDER, M. D., Surgeon to the Massachusetts General Hospital, Out-Patient Department' Assistant in Clinical and Operative Surgery in the Harvard Medical School: assisted by FREDERICK J. COLTON, M. D. Large 8vo, pp. 432, with 585 illustrations. Philadelphia: W. B. Saunders. 1900. [Price, $4.50 net.]

FRACTURES. By CARL BECK, M. D., Visiting Surgeon to St. Mark's Hospital and to the New York German Poliklinik. With an appendix on the Röntgen Rays. Octavo, pp. 335; 178 illustrations. Philadelphia: W. B. Saunders & Co. 1900. [Price, $3.50, net.]

It is unusual to be called upon to prepare notices of two such excellent treatises on fractures published within a few weeks of each other by the same house. But it is a subject that will bear considerable additional material at this time, for it has been some years since a work has appeared upon fractures. We have depended upon what has been said in works on general surgery for description of novel methods that may have been applied to the management of these injuires, and this has been somewhat meager in character. The discovery of the *x*-ray and its application to surgery has contributed considerably to improvements in diagnosis in doubtful cases, and thus methods of treatment have become modified in consequence.

Dr. Scudder, in his treatise, pays great attention to detail, first with reference to diagnosis, and next with regard to treatment. These both are very important. This is a department of surgery that taxes the skill and ingenuity of the surgeon more than any other. Every effort should be put forth to restore a fractured bone to its former usefulness and thus prevent or limit that maiming and unsightliness which result from careless or unskilful treatment, especially of fractures of the extremities.

Perhaps no injury has occasioned more perplexity than Colles's fracture of the radius. It is common, causes maiming and deformity when not properly or adequately treated, and is a constant menace to a surgeon's reputation when unskilfully or imperfectly handled. This author gives most excellent description, advice, treatment and illustrations regarding this troublesome injury. Many other fractures are equally well described and illustrated, but it is unnecessary to enumerate them here. The elbow when injured is another bête noir of the young surgeon, and here will be found much clear instruction relating to it.

The *x*-ray is described in its application to diagnosis of fractures. Massage is another subject of interest in relation to treatment, one perhaps not sufficiently appreciated or insisted upon by surgeons here, but abroad, on the other hand, it is carried to an extreme that would seem unadvisable.

The *x*-ray apparatus is expensive and not easily available outside of hospitals, hence the practical nature of this treatise in giving detailed instructions as to physical examinations, as well as the most elaborate illustrations of dressings will be appreciated by the surgeon in smaller cities and rural centers.

Dr. Beck's work is less pretentious than Scudder's and perhaps not as helpful in many respects. It, however, goes into the application of the *x*-ray more extensively, the author being an enthusiast on the subject. He dedicates his book to Professor Röntgen and classes his discovery as one of the greatest of the age. He asserts in the recognition of fractures "accuracy takes the place of ignorance and doubt, and painful manipulations cease to be necessary for diagnostic purposes."

This author lays stress upon massage when properly employed as an adjunct to treatment. Fixed dressings and the ambulatory treatment are duly considered and accorded their appropriate place in the management of fractures. Beck lacks precision in description in some instances, which detracts from the practical character of this work and might mislead the student or young surgeon. It, however, will be better understood by the surgeon of experience who will not be misled by want of detail. If we may be permitted to express our opinion on this point, we should say that the author's enthusiasm for the *x*-ray had somewhat overshadowed his judgment, hence he has paid too little attention to detailed physical examinations of the injuries with which he deals. The book, however, is a valuable contribution to the literature of surgery and every surgeon will be eager to possess it.

These two books have issued so nearly together that we have ventured to group them in this notice. Not since Hamilton's classic and Stimson's practical working treatise, has anything appeared more welcome to the surgeon who treats fractures than these two books. Each has its special points of excellence, and both will be considered valuable additions to the literature of bone injuries and their treatment. Mechanically, the books are quite similar and are beyond criticism in their dress and illustrations.

International Clinics. A Quarterly of Clinical Lectures and Especially Prepared Articles on Medicine, Neurology, Surgery, Therapeutics, Obstetrics, Pediatrics, Pathology, Dermatology, Diseases of the Eye, Ear, Nose and Throat, etc. Edited by Henry W. Cattell, A. M., M. D., Director of the Ayer Clinical Laboratory of the Pennsylvania Hospital. With the collaboration of John Ashhurst, Jr., M. D., LL. D., and Charles H. Reed, M. D., of Philadelphia; James T Whittaker, M. D., LL. D., of Cincinnati. Vol. II. Tenth Series, 1900. Philadelphia: J. B. Lippincott Company.

This volume is an interesting and instructive continuation of this valuable series, which has become a periodical of considerable proportions and importance. Among the home contributors to this issue we find the names of Edmund Andrews, Robert T. Morris, John B. Murphy, Charles A. Oliver, Edmond Souchon, and J. C. Wilson, living, besides that of Alexander J. C. Skene, recently deceased, all prominent in the literature of medicine. Among the prominent foreign contributors appear the names of J. W. Ballantyne, G. Dieulafoy, H. Senator, and Booth Mackenzie.

The subjects treated of in the book may be classified as follows: therapeutics, seven; medicine, ten; neurology, two; surgery, nine; obstetrics and gynecology, nine; diseases of the eye and ear, eleven; and dermatology, one. Besides these there is a description of the kromskop, by Henry W. Cattell. This is an instrument for reproducing a picture in lights, shades and colors, which was invented by Mr. Frederick E. Ives. The value of such an instrument, made practical, is obvious to every laboratory worker. A kromogram of an ovarian cyst appears as a frontispiece, and it certainly is a beautiful specimen of photography in color.

The book closes with an article entitled, The 1900 meeting of the American Medical Association, by Guy Hinsdale, which is a résumé of a portion of the work done at Atlantic City last June. It is stated that Drs. John Ashhurst, Jr., and James T. Whittaker, both lately deceased, bestowed their last literary work on this book. As associate editors or collaborators, these gentlemen exercised their judgment upon a number of articles, as to the propriety of their appearance in the pages of the clinics. This number well maintains the high standard of excellence already established by this quarterly publication.

PROGRESSIVE MEDICINE. Volume III., September, 1900. A Quarterly Digest of Advances, Discoveries and Improvements in the Medical and Surgical Sciences. Edited by HOBART AMORY HARE, M. D., Professor of Therapeutics and Materia Medica in Jefferson Medical College of Philadelphia. Assisted by CHARLES ADAMS HOLDER, M. D., Assistant Demonstrator of Therapeutics in the Jefferson Medical College. Octavo, pp. 408, with 14 engravings. Issued quarterly. Philadelphia and New York: Lea Brothers & Co. 1900. [Price, $10.00 a year.]

This is one of the most welcome of the contributions to the periodical literature of medicine. It appears quarterly and with great promptitude, a remarkable fact when the great labor of preparing it is considered. The articles in this number are: diseases of the thorax and its viscera, including the heart, lungs and blood-vessels, by William Ewart; diseases of the skin, by Henry W. Stelwagon; diseases of the nervous system, by William G. Spiller; and obstetrics, by William C. Norris.

Dr. Ewart's article deals at considerable length with the diseases which it handles, and will be found particularly attractive to the general practitioner of medicine on account of the many valuable hints on treatment which are found therein. Dr. Stelwagon's article on diseases of the skin is one of the best digests on that subject we have seen and here, again, will be found great aid in treatment, in a most perplexing department of medicine to every one, excepting the trained dermatologist.

Dr. Spiller has presented many important points relating to neurology. Every general practitioner will be interested in what is here said under the head of functional diseases, where Dr. Spiller places those of the unknown pathology, such as epilepsy, hysteria, and the like. Much can be learned from this author about the treatment of these and the neuroses in general.

Perhaps the most attractive section is that on obstetrics, wherein Dr. Norris almost surprises us with the wealth of his contribution. The importance of duly considering the heart in pregnancy is well set forth in this article, and a liberal quotation is made from Dr. Adam H. Wright's recent valuable contribution to the literature of the subject, published in the *American Medical Quarterly*, September, 1899. Fronczak's article in the BUFFALO MEDICAL JOURNAL, January, 1900, on Labordé's method of resuscitating the apparently dead newborn, also receives extended notice. By and large, this is one of the best volumes of this useful series yet issued, and serves to demonstrate the great value of this method of presenting a digest of recent literature.

---

INJURIES TO THE EYE IN THEIR MEDICO-LEGAL ASPECT. By S. BAUDRY, M.D., Professor in the Faculty of Medicine, University of Lille, France, etc. Translated from the original by Alfred James Osterheimer, Jr, M. D., of Philadelphia, Pa. Revised and edited by Charles A Oliver, A. M., M. D., Attending Surgeon to the Wills Eye Hospital; Ophthalmic Surgeon to the Philadelphia Hospital; Member of the American and French Ophthalmological Societies, etc. With an adaptation of the Medico-legal Chapter to the Courts of the United States of America, by Charles Sinkler, Esq., Member of the Philadelphia Bar. Pages, x.—161. Philadelphia: The F. A. Davis Co., Publishers, 1914–16 Cherry street. 1900. [Price, $1.00.]

Great credit should be given those who undertook this work; while concise and brief it represents a tremendous amount of material boiled down for instantaneous reference. The index is very complete and the bibliography gives one an idea of the mass of literature utilised.

Ordinarily the laborious translation and editing of the best of foreign medical literature which enables us to keep in touch with the latest theories and practice abroad has not received anything like the encouragement it should. We trust this book will prove an exception to the rule. Some of the knotty points on expert testimony are well explained.

---

A DICTIONARY OF MEDICINE AND THE ALLIED SCIENCES. Comprising the Pronunciation, Derivation and Full Explanation of Medical, Pharmaceutical, Dental and Veterinary Terms; together with much collateral descriptive matter, numerous tables, etc. By ALEXANDER DUANE, M. D., Assistant Surgeon to the New York Ophthalmic and Aural Institute; Reviser of Medical terms for Webster's International Dictionary. New (3d) edition. In one 8vo volume of 656 pages, with eight full page colored plates. Philadelphia and New York: Lea Brothers & Co. 1900. [Price, $3; full flexible leather $4.00]

The vocabulary of medicine is ever changing, ever increasing, hence new dictionary editions at comparatively short intervals are imperative. This painstaking author has given great study to his work with a view to retain and add the useful and eliminate the obsolete. By studying both sides of this question carefully he has been able to keep his book within convenient limits as to size, while at the same time he has given us a most practical dictionary.

This edition contains much new material which keeps it abreast of the advances in the several departments of medical science. Besides this the work has undergone careful revision so that it may well be said to represent the immediate present. It is one of the most practical of the smaller dictionaries; its letter is plain, its definitions are clear, and its arrangement excellent. We could wish that diphthongs had been omitted more generally, since, in almost every other respect the author has modernised his work. We repeat, however, that it is one of the best dictionaries of its class extant.

A Manual of Obstetrics. By F. A. King, M. D., Professor of Obstetrics and Diseases of Women in the Medical Department of the Columbian University, Washington, D. C., and in the University of Vermont, etc. New (8th) edition. In one 12mo volume of 612 pages, with 264 illustrations. Philadelphia and New York: Lea Brothers & Co., Publishers. 1900. [Price, $2.50 net.]

This excellent manual has so well withstood the test of time, that it needs no extravagant praise to bring it to the attention of those for whom it is intended,—practitioners, instructors and students. While it is elementary in character, it is yet helpful to the three classes named. Professor King is a teacher of great experience as well as an obstetrician of unquestioned skill. These two characteristics serve to make him well qualified to prepare such a manual. In the several editions that have been issued from time to time as they have been demanded he has kept the art he has taught well up to its advances.

This is one of the branches of medical science in which a manual is needful, though, of course, it is not expected to take the place of the systematic treatise. In this edition some changes have been made, a few errors corrected, and forty or more new illustrations supplied. We commend it, if it were possible, even more heartily than ever. It is the obstetric manual, *par excellence.*

